# Role of cell-associated and secretion-independent virulence factors of *Pseudomonas aeruginosa* in microbial keratitis and anti-virulence therapeutic strategies

**DOI:** 10.1007/s10096-026-05504-6

**Published:** 2026-04-08

**Authors:** Tanzina Akter, Shiful Islam, Samea Khan, Kazi Mohammad Zillur Rahman, Mahbuba Akter Lubna, Fiona Stapleton, Mark Willcox

**Affiliations:** 1 School of Optometry and Vision Science, Faculty of Medicine and Health, University of New South Wales (UNSW), Sydney, NSW 2052 Australia; 2 Microbial Biotechnology Division, National Institute of Biotechnology (NIB), Dhaka, 1349 Bangladesh; 3 Department of Biotechnology, Faculty of Natural Science, Norwegian University of Science and Technology, Trondheim, 7034 Norway; 4 School of Biotechnology and Biomolecular Sciences, Faculty of Science, University of New South Wales (UNSW), Sydney, NSW 2052 Australia; 5 Department of Biology, Laboratory of Integrative Biomedicine, University of Copenhagen, Copenhagen, Denmark

**Keywords:** *Pseudomonas aeruginosa*, Microbial keratitis, Virulence factors, Biofilm, Quorum sensing, Anti-virulence therapy

## Abstract

**Purpose:**

Microbial keratitis (MK) caused by *Pseudomonas aeruginosa* is a rapidly progressive and vision-threatening corneal infection worldwide. Even short delays in diagnosis or inappropriate therapy can lead to irreversible corneal scarring and blindness. This opportunistic pathogen colonizes, invades, and damages ocular tissue by taking advantage of disruptions in the corneal epithelial barrier and using a variety of virulence factors. The aim of this study was to summarize the current understanding of *P. aeruginosa*’s cell-associated, and secretion system-independent virulence factors involved in MK and investigate their potential as therapeutic targets.

**Methods:**

A literature search was performed using PubMed, Scopus, Web of Science, MEDLINE, and Google Scholar to review experimental and clinical studies on *P. aeruginosa* keratitis, with a focus on virulence factors, host-pathogen interactions, and emerging anti-virulence strategies. Search terms included combinations of *P. aeruginosa*, microbial keratitis, bacterial keratitis, corneal infection, corneal ulcer, virulence factors, surface- and cell-associated virulence factors, secretion-independent and contact-dependent virulence factors, pathogenicity, and anti-virulence therapies.

**Results:**

Flagella, type IV pili, lipopolysaccharides, outer-membrane proteins, adhesins, biofilm matrix polysaccharides (Psl, Pel, alginate), siderophores, quorum-sensing networks, and secreted metabolites such as pyocyanin may be the key virulence factors in MK. These factors can influence host-pathogen interactions, corneal immunological homeostasis, and encourage drug tolerance and bacterial persistence. Moreover, targeting cell-associated and secretion system-independent virulence mechanisms represents a promising complementary approach along with conventional antimicrobial therapy.

**Conclusion:**

A deeper understanding of the virulence factors may enable the design of targeted therapies. Such approaches could mitigate corneal damage and vision loss while reducing selective pressure for antibiotic resistance.

## Introduction

Microbial keratitis (MK) represents a major cause of corneal blindness worldwide [1–3], particularly in contact lens wearers [4], in tropical and subtropical regions with limited access to prompt ophthalmic care, as well as among patients who are immunocompromised or hospitalized [5, 6]. Among bacterial pathogens, *Pseudomonas aeruginosa* is recognized as one of the most aggressive and destructive causes of MK, often associated with rapid tissue damage, intense inflammation, and poor clinical outcomes leading to blindness within 48–96 h without proper treatment [7].

*P. aeruginosa* possesses a wide range of virulence factors. A virulence factor refers to any bacterial molecule, structure, or trait that enhances the organism’s ability to cause disease in a host [8]. *P. aeruginosa* uses a combination of cell surface-associated structures, secreted metabolites, toxins, effectors and regulatory systems to cause infection [9]. These bacterial machineries are responsible for mediating pathogenesis, which directs the severity of the infection [10, 11].

The cornea is an avascular, immune-privileged tissue in which tightly regulated inflammatory responses are essential for maintaining transparency and visual function. Under normal conditions, *P. aeruginosa* cannot infect an intact cornea; infection is observed only when the epithelial barrier is significantly compromised, either through deep injury reaching the anterior stroma or via direct introduction of bacteria into the stroma [12]. However, it is a highly adaptable opportunistic pathogen and can rapidly exploit breaches in the corneal epithelial barrier. Upon epithelial disruption, this bacterium employs an array of virulence mechanisms to adhere to the corneal surface, compromise epithelial integrity, invade the stroma, and trigger inflammatory responses that ultimately contribute to corneal tissue destruction [13, 14]. Among these virulence mechanisms, cell-associated factors include flagella, type IV pili, fimbriae, surface-associated lipopolysaccharides (LPS), outer-membrane proteins (OMPs), and adhesins, all of which play critical roles in bacterial attachment, motility, and host cell interaction [9]. In contrast, secreted virulence factors are delivered through six distinct secretion systems (type 1 to type 6 secretion systems, T1SS–T6SS), which mediate the release of a variety of toxins and effector proteins [15]. Among these, T3SS associated effectors, ExoU and ExoS are particularly well studied and are associated with distinct disease severity and clinical outcomes in MK [16, 17]. Previous literature on *P. aeruginosa* pathogenesis in MK has focused on secretion system–dependent virulence factors, particularly those delivered through the T1SS to T6SS [15]. Role of the effectors such as ExoU and ExoS from T3SS, as well as secreted proteases including elastase and alkaline protease, other effectors, and toxins have been extensively characterized for their roles in MK and therapeutics drugs against those factors [15].

However, cell-associated and secretion system–independent factors can also play role to disrupt the corneal immune balance by promoting excessive neutrophil recruitment, cytokine release, and sustained inflammation, ultimately exacerbating tissue damage and delaying wound healing [18, 19]. In addition, many of these factors contribute to biofilm formation, antibiotic tolerance, and bacterial persistence, further complicating the management of *P. aeruginosa* keratitis [2]. As such, a thorough understanding of the cell-associated and secretion-independent virulence mechanisms is critical to for a deeper understanding of this bacterium’s ability to infect the eye and the identification of potentially new treatment strategies.

**Fig. 1:**
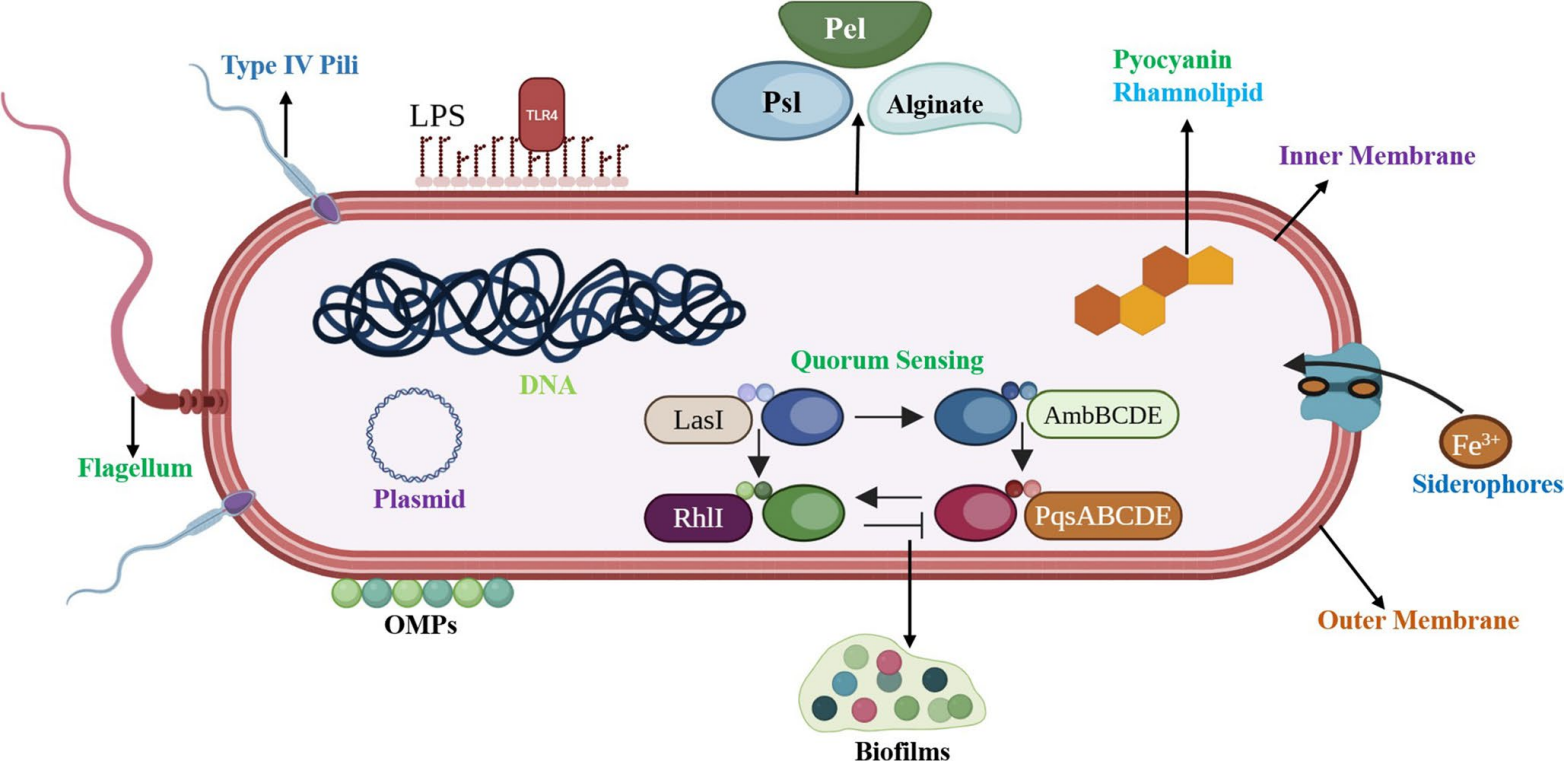
Cell-associated and secretion system-independent virulence factors of *P. aeruginosa* and their associated regulatory systems

This review aims to provide a comprehensive overview of the cell-associated, secretion system-independent virulence factors, virulence regulatory systems of *P. aeruginosa* (Fig. 1) and their roles in the pathogenesis of MK and recent advancements in anti-virulence drug development against the microbe which might be helpful for the development of alternative therapeutic strategies targeting virulence mechanisms.

## Cell surface-associated virulence factors of *P. aeruginosa*

### Flagellum

Flagella are filamentous helical appendages that extend from the bacterial cell wall and are comprised of many repeating protein subunits known as flagellin (FliC), which are encoded by a gene called *fliC* [20]. Flagellin monomers polymerize together to form a long, helical filament that can extend several micrometres from the bacterial cell surface. These filaments are connected to the bacterial cell by a basal body and hook structure that is located within both the inner and outer membranes [21, 22]. The rotation of flagellum is driven by the proton-motive force and is energy dependent. The proper assembly of flagellum also depends on accessory genes such as *fliD*, which encodes the filament cap protein. In *P. aeruginosa*, both *fliC* and *fliD* have been reported to occur as two major allelic variants, designated a- and b-types [23, 24]. Among keratitis isolates, a-type *fliC* has been reported to be more prevalent than b-type *fliC* (76% vs. 24%) [25].

The flagellum of *P. aeruginosa* may contribute to keratitis pathogenesis through two separate mechanisms [19, 20]. First, flagellum-mediated swimming and swarming motilities may allow the bacterium to traverse the viscous tear film and promote access to the corneal surface, particularly at sites of epithelial micro-abrasion or beneath contact lenses. Consistent with this, previous study has reported an association between swimming and swarming behaviours and more severe corneal ulceration, including ring abscess formation, in keratitis [26]. In addition, flagellin has been reported to interact with host surface glycolipids, suggesting that it may facilitate initial attachment and early colonization [18, 26]. However, direct evidence confirming a role of flagella in keratitis models remains to be determined using specific mutants.

Second, the flagellum serves as a pathogen-associated molecular pattern (PAMP). Flagellin is recognized by Toll-like receptor 5 (TLR5) which is expressed on corneal epithelial cells and macrophages [27], and triggers activation of the NF-κB signalling cascade [19]. Once activated, the NF-κB cascade promotes the production and release of pro-inflammatory cytokines such as interleukin-6 (IL-6) and interleukin-8 (IL-8) [18, 19, 28]. This in turn, promotes the recruitment of neutrophils into the infected tissue, which have been shown to be important in tissue destruction as well as pathogen removal [18, 19, 28]. Activation of TLR5 also regulates the production of antimicrobial host defenses [18, 27].

### Type IV Pili

Type IV pili are thin, flexible, filamentous surface appendages that differ structurally and functionally from flagella. They are made up of two main protein subunits known as the major pilin and the minor pilins [29–31]. The major pilin forms the structural core of the pilus of repeating PilA subunits, which are encoded by *pilA*. In contrast, the minor pilins are located at the pilus tip. The minor pilin is essential for pilus extension and adhesion to host cell [30, 32].

The type IV pili help bacterial attachment, twitching motility, biofilm formation, virulence and transfer of genetic material into bacterial cells [29, 33]. Numerous *pil* genes are responsible for the biogenesis of type IV pilus: *pilB* and *pilT* encode ATPases for pilus extension and retraction; *pilC* participates in inner membrane assembly; *pilM*,* pilN*,* pilO*, and *pilP* form the inner-membrane alignment subcomplex; *pilQ* and *pilF* mediate secretion through the outer membrane; and *pilD* is involved in prepilin processing [23, 34, 35]. They allow close interaction with host tissue and microenvironmental adaptation through their dynamic cycles of extension and retraction [29, 33]. The pilus tip contains the minor pilins (*fimU*,* pilV*,* pilW*,* pilX*,* pilE*) and the adhesin *PilY1*, which play roles in pilus initiation, surface sensing, and attachment [36, 37]. Moreover, the initiation and stability of pilus assembly are facilitated by the genes *fimV* and *fimT* [38].

During the early stages of bacterial keratitis, type IV pili facilitate attachment to both the corneal epithelium and to contact lenses [39]. In addition, a type IV pilus mutant was shown to be attenuated in a corneal infection model, providing strong supportive evidence that type IV pili may contribute to virulence. However, this evidence is not definitive, as the study used a non-keratitis strain (PAK) and did not include complementation [39]. Additionally, type IV pili also help in the movement of bacteria between epithelial surfaces and facilitate the development of microcolonies within lens deposits or epithelial crevices [40]. Genotypic and phenotypic analysis of keratitis isolates showed that twitching motility was common and associated with type 3 toxin profiles, supporting a possible role for type IV pili-mediated surface motility in corneal pathogenesis [25]. Furthermore, type IV pili also promote biofilm development, thereby reducing antimicrobial efficacy and supporting persistent infection [41].

### Lipopolysaccharide (LPS)

LPS is a large glycolipid that forms the outer membrane of Gram-negative bacteria. It contains three domains: (1) lipid A acts as an endotoxin and serves as a base for (2) a core oligosaccharide region prior to the (3) distal O-antigen polymer attached at the terminal end. Lipid A is hydrophobic and interacts with the bacteria’s outer membrane and contains a glucosamine disaccharide backbone decorated with N- and O-linked fatty acyl chains. These acyl chains can vary in length and number, and the phosphate groups can be substituted with amino arabinose. This alteration may affect membrane rigidity, resistance to cationic antimicrobial peptides, and modulate recognition by the TLR4-MD2 immune receptor complex [42, 43]. The core oligosaccharide, separated into an inner and outer core, connects lipid A to the O-antigen. The inner core of LPS is relatively conserved and enriched with 3-deoxy-D-manno-oct-2-ulosonic acid and heptoses. These components are essential to LPS assembly and to maintain the integrity of the bacterial membrane [44]. On the other hand, the outer core is composed of glucose and rhamnose which serve as a bridge to support membrane integrity [42]. The O-antigen is composed of a hydrophilic layer made of repeating oligosaccharide subunits that generate extensive amounts of variable-length oligosaccharide chains that extend from the surface of *P. aeruginosa.* The common polysaccharide antigen (CPA or A band), consisting of repeating D-rhamnose units, and the O-specific antigen (OSA or B band), made up of complex heteropolymers containing unique sugars [42, 45–47].This region extends outward from the cell, conferring serotype specificity, mediating immune recognition, and helping the bacterium interact with its environment [42, 43, 45].

LPS of *P. aeruginosa* is more than a structural membrane component, it is a critical factor in barrier function, immune modulation, and virulence. Primarily, it maintains outer-membrane integrity and permeability, protecting the bacterium from environmental stresses such as antibiotics and host antimicrobial peptides [42]. Its role in virulence is many fold. Purified *P. aeruginosa* LPS alone has been shown to induce keratitis [48]. The diversity of the O-antigen polysaccharides allows *P. aeruginosa* to change its surface antigenicity and escape recognition by host antibodies or complement proteins [43, 46]. The oligosaccharide core and O-antigen can mediate adhesion and internalization into corneal epithelial cells during keratitis infection, while variations or modifications in lipid A and O-antigen length alter resistance to antimicrobial peptides [49]. Once colonization is established, lipid A acts as a potent endotoxin and it engages the TLR4-MD-2 complex and CD14 on host immune cells, triggering cytokine production and inflammation [45]. This signalling, as a result, leads to the activation of NF-κB activation, release of cytokines and a neutrophil-rich inflammatory response [50]. TLR4-induced signalling is considered primarily as an innate immunological defence against pathogens, but an excessive or dysregulated signalling response results in the inflammatory damage of corneal tissue in keratitis [27, 50]. A role for LPS in corneal virulence is further supported by in vivo studies showing that rough LPS-deficient (*galU*) mutants are attenuated in murine keratitis models [51] and LPS-specific antibodies reduce corneal disease severity in experimental infection [52]. In addition, LPS may also enhance cell adhesion and biofilm formation by *P. aeruginosa* [42, 53].

### Outer-Membrane Proteins (OMPs) and adhesins

A variety of surface integral outer-membrane proteins (OMPs) and adhesins are present on the outer surface of *P. aeruginosa*. These components differ in size, folding and cellular topology, but together present a multifunctional frontline barrier to the host. The OMPs are generally organized as β-barrel structures which allows the formation of channels or porins for the passage of ions and nutrients. Notable OMPs such as OprF, OprD, and various smaller porins (such as OprG, OprH, OprB) form channels. However, other efflux-associated OMPs (OprM, OprJ, OpmH, OpmD, OpmE) and TonB-dependent transporters (such as FptA, FpvA, FiuA, PiuA, PiuB, PirA) can act as receptors and transporters [54, 55]. In addition to β-barrel proteins, *P. aeruginosa* produces non-fimbrial lectin adhesins, LecA (PA-IL) and LecB (PA-IIL) [56]. These tetrameric carbohydrate-binding proteins are either secreted or remain surface-associated and exhibit specific sugar binding, with LecA recognizing D-galactose and LecB binding L-fucose present on mammalian cells. Additional adhesins, including the Cup family fimbrial systems and the autotransporter EstA, further expand the structural diversity of the *P. aeruginosa* cell surface [56–58].

Core porins such as OprF maintain membrane integrity and transport solute, while specialized channels, including OprD and TonB-dependent transporters, regulate antibiotic entry and nutrient uptake [55, 59]. These OMPs also contribute to cell shape, provide structural stability to the outer membrane, and enhance resistance to environmental stresses such as oxidative damage and antimicrobial exposure [54, 59]. Lectins, particularly LecA and LecB, however, mediate high-affinity carbohydrate-dependent adhesion and promote cell-to-cell aggregation and biofilm matrix formation [57, 60, 61]. Both fimbrial and non-fimbrial adhesins initiate bacterial attachment to host epithelial cells by binding to glycoproteins and glycolipids. As such, this binding allows persistent adhesion during early infection and promotes future invasion [58]. In keratitis pathogenesis, these proteins are likely to contribute to interactions between the bacterium and the ocular surface, although direct in vivo evidence for individual components remains limited.

Clinical strains isolated from eye infections can display increased expression of adhesin/OMP genes, increasing their capacity to attach contact lenses as well as corneal epithelial cells [62]. The major OMPs, including OprF, present extracellular loops that likely mediate close host cell contact and have been experimentally associated with epithelial invasion [40]. *P. aeruginosa* also secretes outer membrane vesicles (OMVs) containing OMPs, adhesins, and enzymes which may allow the bacteria to disseminate virulence factors from a distance [63]. These vesicles can be internalized by corneal epithelial cells and deliver PAMPs that trigger IL-6– and IL-8–mediated inflammatory responses [64, 65]. They have also been shown to induce cytotoxicity in human corneal epithelial cells in vitro and murine corneal epithelium in vivo, while enhancing inflammatory cell recruitment and bacterial adhesion to the corneal surface, thereby contributing to epithelial barrier disruption and helping *P. aeruginosa* evade direct immune attack [63].

## Secondary metabolites – the “pyo”-molecules

### Siderophores

A siderophore is a high-affinity iron-chelating molecule that is secreted extracellularly to scavenge iron from the environment and then imported into the cells when iron availability is limited [66]. Pyoverdine and pyochelin are two types of siderophores produced by *P. aeruginosa.* Both of them chelate ferric iron (Fe³⁺) from the environment in iron limiting conditions, and with the help of specific receptors, deliver them into the bacterial cells [67, 68]. Pyoverdine consists of a fluorescent di-hydroxyquinoline core part that is bound to a small peptide and incorporates hydroxamate and catecholate residues to chelate Fe³⁺ [69]. Pyochelin is a phenolate-containing siderophore without hydroxamate and catecholate groups and binds Fe³⁺ with weaker affinity [70]. Besides scavenging iron, pyoverdine works as a signalling molecule that regulates the expression of other virulence factors, such as proteases and exotoxin A [71]. A comparison of the proteomic profile of a clinical strain from keratitis from a contact lens wearer with a laboratory strain of *P. aeruginosa* found that the clinical strain produced more pyoverdine and hemolysin than the laboratory strain [72]. An ex-vivo study using a PAO1 derived *pvdE* mutant strain (this gene is essential for pyoverdine production) showed that the mutant lost its ability to invade human corneal epithelial cells. Additionally, when mice corneas were infected with *ΔpvdE* strains, no corneal infection was observed demonstrating the significant role of pyoverdine in keratitis [73]. However, complementation studies were not conducted, and therefore definitive effects of *pvdE* mutation cannot yet be made. Pyochelin has yet to be studied for a role in keratitis.

### Pyocyanin

Pyocyanin is a secondary metabolite produced by *P. aeruginosa* in a complex, multi-step process beginning with chorismic acid and is regulated by *phzABCDEFG* operons together with *phzM* and *phzS* genes [74]. Initially, the PhzA-G proteins convert chorismic acid into phenazine-1-carboxylic acid (PCA), which is then methylated by PhzM and hydroxylated by PhzS to yield pyocyanin [75]. This biosynthesis pathway is strictly regulated by quorum sensing (see below) that enables *P. aeruginosa* to synchronize pyocyanin production according to population density [74]. Under anaerobic and microaerophilic conditions pyocyanin acts as electron acceptor and supports *P. aeruginosa* survival by removing excess electrons when the oxidant availability is limited [76]. During corneal infection, pyocyanin may contribute to pathogenesis through multiple mechanisms. It generates reactive oxygen species which disrupt host cell redox homeostasis, depleting intracellular antioxidants such as glutathione, impair immune cell function, and inducing corneal tissue damage [9, 77]. *P. aeruginosa* releases high amounts of pyocyanin during initial colonization to inhibit the growth of other competing microbes within the ocular niche [78]. Additionally, pyocyanin impairs the membrane-associated respiratory chain of other bacteria and reduce their ability to form biofilms. According to one review, approximately 95% of the antimicrobial compounds produced by *P. aeruginosa* for inter-microbial competition are linked to pyocyanin [79]. Collectively, pyocyanin appears to represent a major component of the antimicrobial activity of *P. aeruginosa* against competing bacteria and may contribute to keratitis virulence, but more targeted studies are required [11].

## Virulence regulatory systems

### Quorum sensing

Quorum sensing mediates a myriad of cellular functions including biofilm formation, antibiotic resistance and virulence factor expression [80]. Comprised of four interconnected systems, namely Las, Rhl, PQS and IQS, the quorum sensing system is a cell-density dependent mechanism which enables communication between cells and allows for adaptation to various body environments [81]. Quorum sensing is responsible for regulating up to 10% of the *P. aeruginosa* genome [82].

The Las system encodes the AHL synthase protein, LasI, which subsequently produces autoinducer N-3-oxododecanoyl-L-homoserine lactone (3O-C12-HSL). These N-acyl homoserine lactones (AHLs) are necessary for signal transduction and are disrupted by quenching enzymes AHL acylase, AHL lactonase, and AHL oxidoreductase [83]. This molecule docks at the cognate regulator LasR, thereby controlling the production of virulence factors such as elastase, alkaline protease and exotoxin-A [82]. Importantly, the Las system involves a positive feedback loop, upregulating the production of Rhl and quinolones. The autoinducer synthase RhlI produces *N*-butyryl-L-homoserine lactone (C4-HSL) which binds to corresponding transcriptional regulator RhlR inducing the expression of target genes including *rhlAB*, *lasB*, lectin and other genes necessary for pyocyanin production [82]. The third autoinducer, 2-heptyl-3-hydroxy-4-quinolone, also known as the *Pseudomonas* quinolone signal (PQS) regulates the expression of *lasB* that produces LasB elastase [84]. The synthesis of the PQS molecule is a complex, multistep process requiring *pqsABCDE*,* phnAB*,* pqsR*,* pqsH*, and *pqsL* [85]. The integrating quorum-sensing system (IQS) can mount a response to environmental stressors. There is a dichotomy in the literature as to the synthesis of the IQS molecule (2-(2-hydroxylphenyl)-thiazole-4-carbaldehyde). Although several authors support that the non-ribosomal *ambBCDE* gene cluster is implicated in IQS synthesis [80], there are some who suggest that the IQS molecule is an aeruginaldehyde produced as a result of siderophore pyochelin degradation [86].

When autoinducer concentrations reach a threshold, indicating high bacterial density, LasR- and RhlR-mediated complexes bind promoter regions of target genes, coordinating the expression of extracellular enzymes (e.g., elastase, exotoxin A), proteases, and biofilm components [80]. Feedback regulation exists within these systems: LasR-autoinducer complexes upregulate *lasI* (positive feedback) but also induce RsaL, a repressor that limits *lasI* expression, maintaining balanced virulence factor production [80, 87].

*P. aeruginosa* utilizes quorum sensing (QS) to regulate virulence associated traits and biofilm formation during ocular infection [88–91]. A *lasI* deficient mutant had reduced virulence in an experimental keratitis model [88]. However, the role of *lasR* in keratitis appears to be variable across studies. In one murine keratitis study, a *lasR* mutant remained as virulent as the parental strain, suggesting that LasR was not required for the establishment and maintenance of corneal infection [92]. In contrast, another study showed that an isogenic Δ*lasR* mutant in a rabbit keratitis model supported the involvement of quorum sensing in bacterial proliferation and corneal perforation [93]. Interestingly, clinical studies have shown that 21.8% of keratitis isolates harbour loss-of-function *lasR* mutations but these may be associated with worse clinical outcomes despite altered QS activity [94, 95].

### Biofilm formation

Biofilm is a complex microbial community that adhere to a stable surface and embed in an exopolysaccharide matrix [96]. A number of virulence factors, such as polysaccharide (Pel, Psl and alginate), rhamnolipids, type IV pili, flagella, LPS and pyocyanin are involved in successful biofilm formation in *P. aeruginosa* [26]. Biofilms may subsequently contribute to more severe and persistent keratitis by limiting antimicrobial penetration through the extracellular matrix and by altering bacterial gene expression and physiology, thereby increasing tolerance to host defences and antimicrobial agents and delaying recovery [26]. In a recent study with an ex-vivo porcine keratitis model, biofilm-embedded bacteria showed markedly reduced susceptibility to commonly used ophthalmic antibiotics such as fluoroquinolones and aminoglycosides, complicating clinical management [97]. A study using *P. aeruginosa* clinical isolates from the double-blind Steroids for Corneal Ulcers Trial (SCUT) found that biofilms are associated with worse visual prognosis upon presentation and three months post-treatment [98].

*P. aeruginosa* readily forms biofilms on contact lenses, and these biofilms significantly increase the risk and severity of contact-lens-associated keratitis [40]. Biofilms can form both on the corneal surface and the lenses, creating a safe niche for bacterial hideout [40, 99]. Clinical isolates collected from contaminated lenses and lens solutions are commonly strong biofilm-formers and also multidrug-resistant [100]. If contact lenses are already contaminated with *Pseudomonas* biofilms, the lens wearer may be at an increased risk for *Acanthamoeba* (a protozoan) infection as well [101]. Besides, biofilm promotes and enables accumulation of bacterial proteases and elastases, which contribute to corneal tissue degradation and ulceration [102].

#### Polysaccharide Synthesis Locus (Psl)

The polysaccharide synthesis locus (Psl), is a penta-saccharide of D-mannose, D-glucose, and L-rhamnose monomers that is implicated in biofilm development [103]. Psl assists in anchoring of bacteria to surfaces and neighbouring cells [103]. As *P. aeruginosa* moves, it leaves behind Psl trails that promote further cell movement on surfaces as well as microcolony formation [104]. Psl also stimulates biofilm growth by increasing cyclic di-guanosine monophosphate levels [105]. In mature biofilms, Psl strengthens the structure by linking CdrA (cyclic di-GMP-regulated adhesin A) and extracellular DNA [106, 107]. Practically, Psl limits antibiotic penetration and weakens host immune responses by reducing complement deposition and inhibiting neutrophil activity [108, 109]. Although analysis of isolates from the SCUT revealed that nearly all *P. aeruginosa* keratitis strains expressed the Psl exopolysaccharide [98], it was not strictly necessary for biofilm formation particularly in the presence of other EPSs such as pellicle (Pel) or alginate, and surface appendages including flagellum and pili which are useful in biofilm formation [106, 110]. Indeed, significant heterogeneity has been identified between non-mucoid strains which may utilise either Psl or Pel as the primary structural polysaccharide of the biofilm matrix [110]. Dependence of biofilm formation on the Pel and Psl polysaccharides is strain-dependent. For example, common laboratory strain PAO1 is primarily Psl-dependent, whereas PA14, which lacks essential *psl* biosynthetic genes, relies predominantly on Pel for biofilm matrix [106, 110].

#### Pellicle (Pel)

Pel, a cationic polymer composed of galactosamine and N-acetyl galactosamine residues, is primarily associated with non-mucoid strains of *P. aeruginosa* and plays a crucial role in biofilm formation, particularly pellicle formation at the air-liquid interface [111]. Its positive charge allows cross-linking between negatively charged extracellular DNA (eDNA) through the formation of ionic bonds [112]. Moreover, its cationic nature imparts resistance to positively charged antibiotics such as tobramycin and further renders nuclease-based therapies ineffectual by halting the digestion of eDNA by DNase I [113]. Until now, no studies have specifically addressed the role of Pel in the context of keratitis, underscoring the necessity for additional research.

#### Alginate production

*P. aeruginosa* can synthesise alginate, an anionic linear exopolysaccharide (EPS) composed of 3-D-mannuronic acid and the C-5 epimer C~-L-guluronic acid [114]. EPS are integral to the initial attachment and anchoring of *P. aeruginosa* to solid surfaces [115]. Alginate can be a primary component of *P. aeruginosa* biofilms and plays a protective role by creating reduced diffusion of nutrients, pH and waste materials [107]. Alginate producing strains form comparatively thicker and more viscous biofilms than the non-producing strains. Moreover, acetylated uronic acids in alginate enhance water retention, thereby preventing dehydration and desiccation of bacterial cells [107, 116]. A study from Iran reported that *P. aeruginosa* from keratitis, produced biofilms and harboured *algD* gene (that synthesises alginate) suggesting that alginate may be play a role in *P. aeruginos*a keratitis [117]. However, more research is needed to definitively demonstrate such a role, by for example, comparing *algD* mutants to parent strains in animal models of keratitis.

#### Rhamnolipid

*Pseudomonas* Rhamnolipids are a class of glycolipids consisting of sugar L-rhamnose (head) and one or two molecules of 3-hydroxy fatty acids (tail). Head and tail units are joined via an O-glycosidic linkage [118]. They are synthesized by *rhlA*,* rhlB*, and *rhlC* genes under the control of Las and Rhl, two interrelated quorum sensing systems in *P. aeruginosa* [119]. Rhamnolipids are important for biofilm architecture and maintenance [120], for the detachment of *P. aeruginosa* cells from biofilms [121] as well as for swarming motility on semi-solid surfaces [122, 123]. In keratitis, rhamnolipids contribute to biofilm formation by reducing surface tension and keeping non-colonized channels open within the biofilm structure, which helps the bacteria persist [118, 120]. Their ability to reduce surface tension supports bacterial motility, including swarming, and in iron-limited conditions, they enhance twitching motility. *P. aeruginosa* uses rhamnolipids to suppress the host’s innate immune defence [118]. Rhamnolipids were found to suppress the host’s immune response by preventing the pathogen induced activity of human β-defensin 2, thereby impeding the body’s ability to combat the infection [124].

## Therapeutic targets against cell-associated and secreted virulence factors

As the antimicrobial resistance in *P. aeruginosa* is increasing [125–127], there is growing interest in development of anti-virulence strategies targeting bacterial pathogenic mechanisms rather than bacterial viability. Several cell-associated and virulence factors, independent of classical secretion systems, have emerged as promising therapeutic targets in *P. aeruginosa* keratitis (Table 1). These approaches aim to attenuate bacterial virulence, limit tissue damage, and reduce selective pressure for resistance development.

Bacteriophages, vaccines, and antibiotics targeting the outer membrane components such as OprF, and OprI have been explored as therapeutic targets. Use of intramuscular vaccines targeting OprF/OprI-based have progressed to Phase II clinical trials [128]. Moreover, antibiotics such as pentamidine have demonstrated early-phase clinical potential against the outer membrane proteins of the microbe [9, 129, 130]. During corneal infection, surface components such as LPS, flagella/flagellin, and type IV pili may contribute to corneal adhesion, motility, and innate immune activation in experimental keratitis models [39, 51, 131, 132], although direct evidence for a role of fimbriae in keratitis remains to be tested. Therapeutic antibodies against LPS have advanced to Phase III clinical trials [45, 133, 134], while bacteriophage-based approaches targeting flagella are under phase III [134–136] and pili and fimbriae are under active investigation [134, 137, 138]. These strategies aim to disrupt initial stage of infection preventing bacterial attachment and colonization on the corneal surface. To date, none of these have been specifically tested for efficacy in keratitis models.

Biofilm-associated virulence factors represent another important therapeutic focus, as biofilm formation leads to treatment failure and persistent of infection in keratitis. Agents such as Oligo-G (uluronic acid), which interfere with biofilm structure and motility, have reached Phase II trials [139]. Degradation of biofilm matrix components, including Psl and Pel polysaccharides, by enzymes (PslG and PelA), has shown promising preclinical results [140]. Furthermore, targeting siderophore-mediated iron acquisition using antibiotic–siderophore conjugates represents an emerging strategy to limit bacterial survival in the nutrient-restricted ocular environment [66]. Collectively, these anti-virulence approaches highlight alternative and adjunctive therapeutic strategies against *P. aeruginosa* which might be promising treatment for keratitis, although as yet no studies have examined their efficacy.

**Table 1:** Therapeutics targets against *P. aeruginosa’s* cell associated and secreted virulence factors

Therapeutic targets	Therapeutic	Development status	References
OprF/OprI	IM vaccine	Phase II	[128]
Outer Membrane Proteins	Antibiotics (Pentamidine)	Phase I	[9, 129, 130]
Lipopolysaccharide	Antibody	Phase III	[45, 133, 134]
Flagella	Bacteriophages	Phase III	[134–136]
Pili and Fimbriae	Phages	Not mentioned	[134, 137, 138]
Biofilms and motility	Oligo-G (uluronic acid)	Phase II	[139]
Psl and Pel matrix polysaccharides	Enzyme (PslG, PelA)	Preclinical	[140]
Siderophores	Antibiotic-siderophore	Not mentioned	[66]

## Conclusion

Virulence in *P. aeruginosa* keratitis is the result of multiple interacting factors rather than a single dominant mechanism. Cell surface components such as flagella, type IV pili, LPS, and outer-membrane proteins drive early corneal colonization, epithelial invasion, and host inflammatory responses. These early events often lead to tissue damage, especially when inflammation becomes excessive or prolonged. In chronic infection, antibiotic-resistant conditions are further enhanced by the formation of biofilms, where exopolysaccharides such as alginate and Psl serve essential structural and defensive functions. Simultaneously, quorum-sensing systems and siderophore-mediated iron uptake regulate virulence factors expression and metabolic adaptation, increasing infection severity and persistence.

The growing problem of multidrug resistance highlights the need for anti-virulence therapies. Targets such as outer-membrane proteins, LPS, flagella, pili, biofilm matrix polysaccharides (Psl, Pel, alginate), siderophores, and quorum sensing pathways are actively being explored. Several phages, vaccines, enzymes, and small-molecule therapies have shown promise in preclinical and early clinical studies which needs to be continued for developing more effective, targeted interventions aimed at preserving corneal integrity and visual function.

## Data Availability

Data is provided within the manuscript.
